# Blue LED-pumped intense short-wave infrared luminescence based on Cr^3+^-Yb^3+^-co-doped phosphors

**DOI:** 10.1038/s41377-022-00816-6

**Published:** 2022-05-13

**Authors:** Yan Zhang, Shihai Miao, Yanjie Liang, Chao Liang, Dongxun Chen, Xihui Shan, Kangning Sun, Xiao-Jun Wang

**Affiliations:** 1 Key Laboratory for Liquid-Solid Structure Evolution and Processing of Materials, Ministry of Education, Shandong University, Jinan, 250061 China; 2 Jiangsu Bree Optronics Co., Ltd, Nanjing, 211103 China; 3 Department of Physics, Georgia Southern University, Statesboro, GA 30460 USA

**Keywords:** Inorganic LEDs, Photonic crystals

## Abstract

The growing demand for spectroscopy applications in the areas of agriculture, retail and healthcare has led to extensive research on infrared light sources. The ability of phosphors to absorb blue light from commercial LED and convert the excitation energy into long-wavelength infrared luminescence is crucial for the design of cost-effective and high-performance phosphor-converted infrared LEDs. However, the lack of ideal blue-pumped short-wave infrared (SWIR) phosphors with an emission peak longer than 900 nm greatly limits the development of SWIR LEDs using light converter technology. Here we have developed a series of SWIR-emitting materials with high luminescence efficiency and excellent thermal stability by co-doping Cr^3+^-Yb^3+^ ion pairs into Lu_0.2_Sc_0.8_BO_3_ host materials. Benefitting from strong light absorption of Cr^3+^ in the blue waveband and very efficient Cr^3+^→Yb^3+^ energy transfer, the as-synthesized Lu_0.2_Sc_0.8_BO_3_:Cr^3+^,Yb^3+^ phosphor emits intense SWIR light in the 900–1200 nm from Yb^3+^ under excitation with blue light at ~460 nm. The optimized phosphor presents an internal quantum yield of 73.6% and the SWIR luminescence intensity at 100 °C can still keep 88.4% of the starting value at 25 °C. SWIR LED prototype device based on Lu_0.2_Sc_0.8_BO_3_:Cr^3+^,Yb^3+^ phosphor exhibits exceptional luminescence performance, delivering SWIR radiant power of 18.4 mW with 9.3% of blue-to-SWIR power conversion efficiency and 5.0% of electricity-to-SWIR light energy conversion efficiency at 120 mA driving current. Moreover, under the illumination of high-power SWIR LED, covert information identification and night vision lighting have been realized, demonstrating a very bright prospect for practical applications.

## Abstract (web summary)

Cr^3+^-Yb^3+^-co-doped phosphors that emit intense short-wave infrared light in the 900–1200 nm upon blue light excitation have been developed.

## Graphical abstract

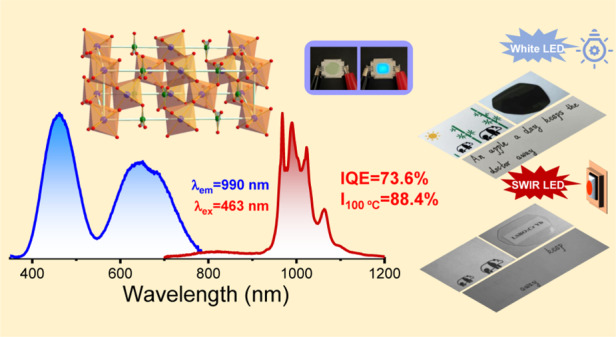

## Introduction

Short-wave infrared (SWIR, 900–1700 nm) light sources have aroused widespread interest for a wide range of applications in the fields of night vision surveillance, optical communications, remote sensing, and biomedical imaging^1–7^. Recent advancement in smart and portable SWIR spectroscopy for product sorting, quality checking, and noninvasive health monitoring also necessitates the development of high-performance and cost-effective SWIR light emitters^8–10^. The specific application requirement determines the research interest of illuminator in the SWIR spectral region, which correlates with the unique characteristics of SWIR light^11,12^, such as being unlikely to be seen by standard detectors, low absorption and scattering by biological tissue, and high penetration power in complex environmental conditions (haze, fog, smoke, or dust in day and night).

In contrast to traditional light sources (tungsten filament and halogen lamps), infrared LEDs based on III-V inorganic semiconductors (e.g., GaAs and InGaAs) are replacing these unwieldy and low-efficiency lamps for use in smart SWIR devices owing to their solid-state nature, compact design, low power consumption and high radiant flux within 1050–1650 nm (e.g., 750 mW@1000 mA, Ushio EDC1050GD-1100)^13,14^. However, the inherent limitations of SWIR LED chips such as complex processing requirements, relatively high product price, and a narrow band of SWIR light (<50 nm), also make them difficult to meet the wide application for daily use. Very recently, solution-processed semiconductors emitting in the SWIR (e.g., organics, perovskites, or colloidal QDs), are offering new and exciting possibilities for SWIR LEDs^15–20^. Nevertheless, organic and perovskite LEDs usually exhibit poor device performance with low external quantum efficiency at wavelengths >950 nm^21,22^. QD LEDs can exhibit longer-wavelength emissions, spanning a large part of the SWIR region with emissions up to 1500 nm, but challenges remain in achieving high and stable power output, reducing the roll-off of external quantum efficiency, extending the operation lifetime, and avoiding the presence of toxic elements^22^.

On the other hand, converter technology has recently become a cost-effective and promising approach to design and develop high-performance solid-state SWIR light sources, which combine commercial high-power LED chips with quantum dots or inorganic phosphors doped with transition metals or trivalent lanthanides, as the device mechanism occurs in phosphor-converted white and near-infrared LEDs^23–26^. For example, Wang et al. fabricated efficient infrared LEDs with a peak wavelength at 980 nm by using PbSe QDs as the light converter of blue GaN LED and reached an external quantum efficiency of 5.3%^27^. Pradhan et al. demonstrated an optical output power of 14 mW with 13% of power conversion efficiency at a driving current of 360 mA from broadband SWIR light emitters based on stacks of multi-bandgap PbS QDs and 595 nm LED^28^. Fu and co-workers assembled an infrared LED prototype device by integrating SrO:Er^3+^ phosphors with 380 nm UV-emitting chip, achieving 1.54 μm SWIR luminescence due to the characteristic electron transition of Er^3+,^^29^. Pichon et al. designed LED-pumped SWIR luminescent concentrator by combining 940 nm LEDs with Yb,Er co-doped glass, which emits at 1550 nm with an optical conversion efficiency of 1.29% at 600 mA^30^. Wang et al. reported a SWIR LED device by coating 450 nm blue LED chip with Zn_1.5_Sn_0.5_Ga_1.0_O_4_:Cr^3+^,Ni^2+^ phosphors, which presented a broad emission band over 1100–1650 nm with an emission peak at 1330 nm^31^. Recently, Miao et al. demonstrated a SWIR output power of 4.78 mW with an 1120 nm emission peak and power conversion efficiency of 4.4% at 60 mA by employing LiScGeO_4_:Cr^3+^ phosphors as the blue-light converter^32^.

Trivalent ytterbium (Yb^3+^) is a favorable luminescent center, which can emit SWIR light in the 940–1200 nm with a peak maximum at ~1000 nm due to the ^2^F_5/2_ → ^2^F_7/2_ electron transition^33^. As a result, Yb^3+^-activated luminescent materials have been paid much more attention for optoelectronic applications in the SWIR, including solid-state lasers^34^, photon upconversion^35–37^, spectral converters for Si photovoltaics^38,39^ and SWIR LED devices^6,17^. Nevertheless, the blue light absorption ability of Yb^3+^ is significantly weak due to the absence of energy levels in the blue region, making it hard to achieve high-efficient SWIR luminescence by the use of readily available and high-power blue LED chips. Just recently, Yb^3+^ has been co-doped into Cr^3+^-activated broadband NIR-emitting phosphors to broaden the spectral bandwidth and improve the NIR luminescence performance in view of the efficient Cr^3+^→Yb^3+^ energy transfer under the excitation of blue LED^40–45^. However, with regard to this application, Cr^3+^ emission and Yb^3+^ emission usually appear simultaneously with comparable luminescence intensities in order to achieve super broadband NIR LED application, while the intense and dominant emission band over 940–1200 nm from Yb^3+^ for SWIR LEDs and their practical applications have largely gone unnoticed.

Here in this work, we show that Lu_0.2_Sc_0.8_BO_3_:Cr^3+^,Yb^3+^ is a promising SWIR phosphor, in which Cr^3+^, as a sensitizer, strongly absorbs blue LED emission and efficiently transfers the excitation energy to Yb^3+^ at a high concentration, giving rise to intense SWIR luminescence peaking at ~1000 nm in a wavelength range of 940–1200 nm. Spectral analysis results indicate that Lu_0.2_Sc_0.8_BO_3_:Cr^3+^,Yb^3+^ phosphor has high luminescence efficiency and very good stability against heat, suggesting it has great potential for phosphor-converted SWIR LEDs.

## Results

### Structural characterization of Lu_0.2_Sc_0.8_BO_3_:Cr^3+^,Yb^3+^ phosphors

**Fig. 1:**
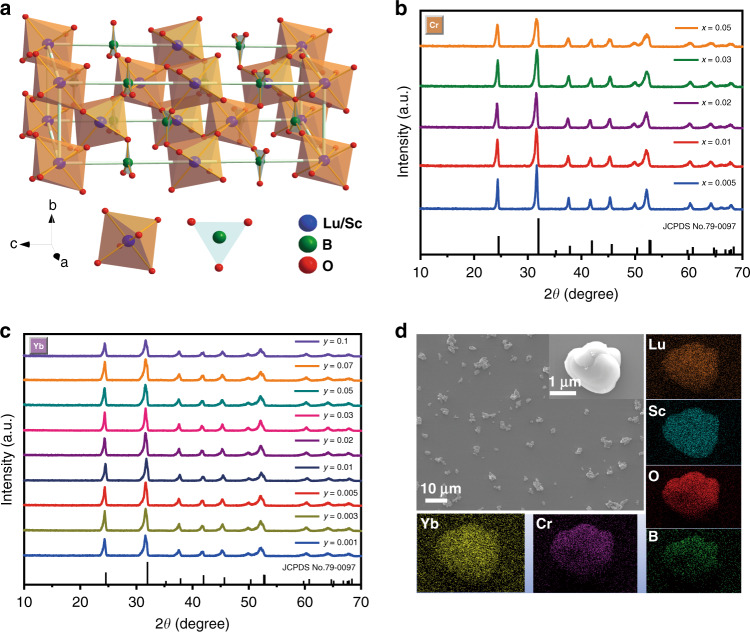
Crystal structure and morphology. **a** Crystal structure of Lu_0.2_Sc_0.8_BO_3_ compound and the coordination diagrams of trivalent cations. **b**, **c** XRD patterns of Lu_0.2_Sc_0.8_BO_3_:*x*Cr^3+^ (*x* = 0.005–0.05) and Lu_0.2_Sc_0.8_BO_3_:2%Cr^3+^,*y*Yb^3+^ (*y* = 0.001–0.1). **d** SEM and corresponding elemental mapping of Lu_0.2_Sc_0.8_BO_3_:2%Cr^3+^, 5%Yb^3+^ phosphor

**Fig. 2:**
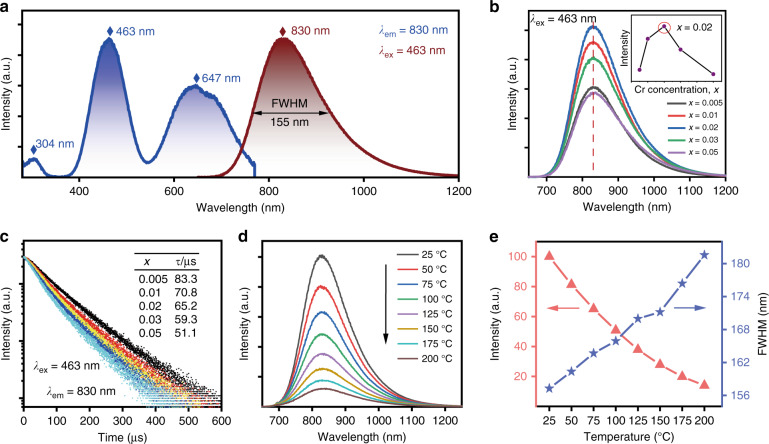
Luminescence properties of Lu_0.2_Sc_0.8_BO_3_:Cr^3+^ phosphors. **a** Normalized excitation and emission spectra of Lu_0.2_Sc_0.8_BO_3_:Cr^3+^. **b** Dependence of emission spectra of Lu_0.2_Sc_0.8_BO_3_:*x*Cr^3+^ (*x* = 0.005–0.05) on the Cr^3+^ concentration. Inset: the changes of integral luminescence intensity at varied Cr^3+^-doping concentration. **c** Luminescence decay curves of Lu_0.2_Sc_0.8_BO_3_:*x*Cr^3+^ phosphors. **d** Emission spectra of Lu_0.2_Sc_0.8_BO_3_:2%Cr^3+^ phosphor at different temperature from 25 to 200 °C. **e** NIR emission intensity and FWHM of Lu_0.2_Sc_0.8_BO_3_:2%Cr^3+^ phosphor at different temperature

The schematic crystal structure of Lu_0.2_Sc_0.8_BO_3_ is displayed in Fig. 1a, which crystallizes in the rhombohedral crystal system in R$\overline 3$c space group^46^. The trivalent cation of Lu/Sc is coordinated with six O^2−^ to constitute [LuO_6_]/[ScO_6_] octahedron, while B atom is connected by three O atoms to form a [BO_3_] triangle. The connection between octahedrons and [BO_3_] groups occurs only through corner-sharing in Lu_0.2_Sc_0.8_BO_3_ solid solution. The [LuO_6_]/[ScO_6_] layers and [BO_3_] groups are arranged alternately. Since six-coordinated Lu^3+^ and Sc^3+^ ions have ionic radii of 0.861 Å and 0.745 Å, respectively, the incorporation of larger Lu^3+^ into ScBO_3_ will lead to the distortion of the crystal structure^46^. X-ray diffraction (XRD) patterns of Lu_0.2_Sc_0.8_BO_3_:*x*Cr^3+^ (*x* = 0.005–0.05) and Lu_0.2_Sc_0.8_BO_3_:2%Cr^3+^,*y*Yb^3+^ (*y* = 0.001–0.1) samples are presented in Fig. 1b, c, respectively. All the XRD patterns can be indexed to the calcite phase of ScBO_3_ (JCPDS No. 79–0097), which indicate that introducing Cr^3+^ and Yb^3+^ dopants will not cause the formation of the impurity phase. However, in comparison with ScBO_3_ crystal, the diffraction peaks of the as-synthesized phosphors show a significant shift to the lower angle region, revealing that the Lu^3+^ ion with a larger ionic radius has been dissolved into the ScBO_3_ lattice and Lu_0.2_Sc_0.8_BO_3_ solid solution has been formed. Furthermore, as shown in Fig. 2b, the diffraction peak at ~31.6° becomes progressively asymmetric and all peaks seem to get broader as Cr^3+^ concentration increases, which probably results from the fact that the octahedra gets progressively distorted since the ionic radius of Cr^3+^ with six-coordination number (0.615 Å) is smaller than that of trivalent cation (Lu^3+^/Sc^3+^). The Rietveld refinement of Lu_0.2_Sc_0.8_BO_3_:2%Cr^3+^,5%Yb^3+^ phosphor is also presented in Fig. S1. The observed diffraction peaks are in good agreement with the simulated counterparts with *R*wp value of 8.84% and *R*p value of 6.27%, indicating the refinement results are accurate. Detailed crystal structure parameters and cell parameter values are presented in Table S1.

In order to further study the microstructure and element composition of Lu_0.2_Sc_0.8_BO_3_:Cr^3+^,Yb^3+^ phosphors, SEM images and EDS element mapping of the Lu_0.2_Sc_0.8_BO_3_:2%Cr^3+^ and Lu_0.2_Sc_0.8_BO_3_:2%Cr^3+^,5%Yb^3+^ are obtained, as presented in Fig. 1d and Fig. S2, respectively. SEM imaging in Fig. 1d reveals an irregular shape of the as-synthesized phosphor particles with particle sizes ranging over 2–5 μm. The smooth surface of particles also suggests the high crystallinity of the obtained phosphors. The EDS elemental mapping results demonstrate that Lu, Sc, B, Cr, Yb, and O are uniformly dispersed throughout the obtained phosphor particle. ICP-MS results further verify that both Cr^3+^ and Yb^3+^ emitters have been introduced into Lu_0.2_Sc_0.8_BO_3_ lattice (Table S2).

### Luminescence properties of Lu_0.2_Sc_0.8_BO_3_:Cr^3+^ phosphors

The photoluminescence properties of Lu_1−*z*_Sc_*z*_BO_3_:Cr^3+^ phosphors (z = 0.2–0.8) with different Lu/Sc ratios are shown in Fig. S3. As the Sc^3+^ content increases, the NIR emission intensity of the as-synthesized phosphor shows a gradual increase and reaches a maximum at *z* = 0.8. Moreover, Lu_0.2_Sc_0.8_BO_3_:Cr^3+^ phosphor exhibits the optimum luminescence thermal stability (Fig. S4). The excitation spectrum is composed of three evident excitation bands by monitoring at 830 nm emission, as depicted in Fig. 2a, which can be assigned to the electron transitions of Cr^3+^ emitters, including the 304 nm excitation peak resulting from the Cr^3+ 4^A_2_ → ^4^T_1_(^4^P) transition, the 463 nm excitation peak resulting from the Cr^3+ 4^A_2_ → ^4^T_1_(^4^F) transition and the 647 nm excitation peak resulting from the Cr^3+ 4^A_2_ → ^4^T_2_(^4^F) transition, respectively. Under 463 nm blue light excitation, Lu_0.2_Sc_0.8_BO_3_:Cr^3+^ phosphors emit NIR light with a peak maximum at ~830 nm and an FWHM of ~155 nm, which can be attributed to the electron transition from the ^4^T_2_(^4^F) exciting level to the ^4^A_2_ ground state level. Moreover, the NIR luminescence spectra of Lu_0.2_Sc_0.8_BO_3_:Cr^3+^ phosphor remain almost unchanged even at 77 K, as depicted in Fig. S5. It is well known that the Tanabe–Sugano diagram can directly manifest the energy level distribution of Cr^3+^ located in octahedral site. Therefore, the corresponding crystal field parameters of Lu_0.2_Sc_0.8_BO_3_:Cr^3+^ are calculated according to the following formulas^47,48^:

1 $$10D_q = E\left({{}^4}T_2 \right) = E\left({{{}^4}{{{{\mathrm{A}}}}_2} \to {{}^4}{{{{\mathrm{T}}}}_{2}}} \right)$$

2 $$\frac{{D_q}}{B} = \frac{{15 \times \left( {\frac{{\Delta E}}{{D_q}} - 8} \right)}}{{\left( {\frac{{\Delta E}}{{D_q}}} \right)^2 - \frac{{10\Delta E}}{{D_q}}}}$$

3 $${\it{\Delta }}E = E\left( {{}^4{{{\mathrm{T}}}}_1} \right) - E\left( {{}^4{{{\mathrm{T}}}}_2} \right) = E\left( {{}^4{{{\mathrm{A}}}}_2 \to {}^4{{{\mathrm{T}}}}_1} \right) - E\left( {{}^4{{{\mathrm{A}}}}_2 \to {}^4{{{\mathrm{T}}}}_2} \right)$$

where *E*(^4^T_2_) represents the energy position of ^4^T_2_ energy level, *E*(^4^T_1_) is the energy position of ^4^T_1_ energy level, *B* is the Racah parameter, *D*_*q*_ is the crystal field parameter. Therefore, the *D*_*q*_/*B* value of Lu_0.2_Sc_0.8_BO_3_:Cr^3+^ is calculated to be 2.51, as shown in Fig. S6. Moreover, the two main excitation peaks of Lu_0.2_Sc_0.8_BO_3_:Cr^3+^ are close to the values in the ScBO_3_:Cr^3+^ phosphor reported by Fang et al. (*D*_*q*_/*B* = ~2.88)^49^. Generally, when Cr^3+^ ions reside in a strong crystal field (i.e., *D*_*q*_/*B* > 2.3), the narrowband luminescence assigned to the ^2^E → ^4^A_2_ transition occurs in this system. However, even though the obtained *D*_q_/*B* value in Lu_0.2_Sc_0.8_BO_3_ host is much larger than 2.3, the Cr^3+^ emitter suffers from a weak crystal field and presents a NIR broadband emission. This inconsistency has also been noted in some other oxide-based NIR phosphors^50–53^, which is probably attributed to the distortion of the octahedron, while the classic Tanabe–Sugano diagram is on the basis of the nearly perfect octahedral position of Cr^3+^ ^42,54^. Actually, an inspection of the crystal structure of Lu_1-x_Sc_x_BO_3_ crystal shows that trivalent metal cations are located only in one site that is not perfectly octahedral, but is distorted to a lower local S_6_ symmetry^55^. Moreover, for Lu_1-x_Sc_x_BO_3_ solid solution, it is demonstrated by Liang et al. that the distortion ratio of [LuO_6_]/[ScO_6_] octahedra becomes larger as the content of Sc^3+^ increases in Lu_1-*x*_Sc_*x*_BO_3_ lattice and the distortion ratio reaches a maximum when *x* = 0.7 (Lu_0.3_Sc_0.7_BO_3_)^46^.

The emission spectra of Lu_0.2_Sc_0.8_BO_3_:Cr^3+^ samples with different Cr^3+^ doping concentrations upon excitation with blue light are depicted in Fig. 2b. The emission peak and spectral distribution of luminescence spectra basically keep constant with varying Cr^3+^ content from 0.005 to 0.05. But the NIR luminescence intensity shows an increasing trend from x = 0.005 to 0.02 and then falls off when the Cr^3+^ content continues to increase (the upper inset in Fig. 2b). Along with increasing the amount of Cr^3+^ emitters, the energy transfer between the Cr^3+^ ions may end up in the defect-related centers, and this probability increases at high Cr^3+^ concentrations^56^. These killer centers relax the excitation energy to the lattice via a nonradiative process, which leads to the concentration quenching of luminescence^57^. Meanwhile, the luminescence decay curves of Lu_0.2_Sc_0.8_BO_3_:*x*Cr^3+^ phosphors in Fig. 2c can be fitted with a single exponential function:

4 $$I\left( t \right) = I_0exp( - t/\tau )$$

where *I*(*t*) represents the NIR luminescence intensity at time *t*, *I*_*0*_ is the initial intensity, and *τ* is the lifetime. The slight deviation from the single exponential term is assigned to the energy transfer between the Cr^3+^ ions and their random distribution among the octahedral sites, especially under high doping concentrations. The fluorescence lifetime of Lu_0.2_Sc_0.8_BO_3_:Cr^3+^ sample decreases from 83.3 to 51.1 μs as the Cr^3+^ concentration increases from 0.005 to 0.05. To investigate the temperature-dependent NIR photoluminescence of Lu_0.2_Sc_0.8_BO_3_:Cr^3+^, emission spectra at a different temperatures from 25 to 200 °C are depicted in Fig. 2d. With increasing the test temperature, the NIR emission peak and spectral shape do not vary with temperature, but the luminescence intensity shows an obvious decrease trend owing to the thermal quenching mechanism. At 100 and 150 °C, the integral emission intensities decline to 50% and 27.9% of the starting values at 25 °C, respectively. Moreover, NIR emission from higher excited states leads to the broadened spectral width from 157 to 182 nm with temperature elevating, as presented in Fig. 2e. In addition, the photoluminescence quantum yield of Lu_0.2_Sc_0. 8_BO_3_:2%Cr^3+^ is measured to be 26.1% when exciting with 463 nm light (Fig. S7a).

### SWIR photoluminescence properties of Lu_0.2_Sc_0.8_BO_3_:Cr^3+^,Yb^3+^ phosphors

Lu_0.2_Sc_0.8_BO_3_:Cr^3+^ phosphor exhibits decent NIR photoluminescence performance considering its relatively short peak wavelength (<850 nm), poor thermal stability, and low absolute quantum efficiency, which will greatly limit its practical application for NIR spectroscopy. On the other hand, Yb^3+^ ion can generate desirable SWIR emission at ~1000 nm, but it usually does not have characteristic absorption in the blue region. Considering the efficient Cr^3+^→Yb^3+^ energy transfer, Yb^3+^ ions are co-doped into the Lu_0.2_Sc_0.8_BO_3_:2%Cr^3+^ phosphors to improve the photoluminescence performance and extend their applications.

**Fig. 3:**
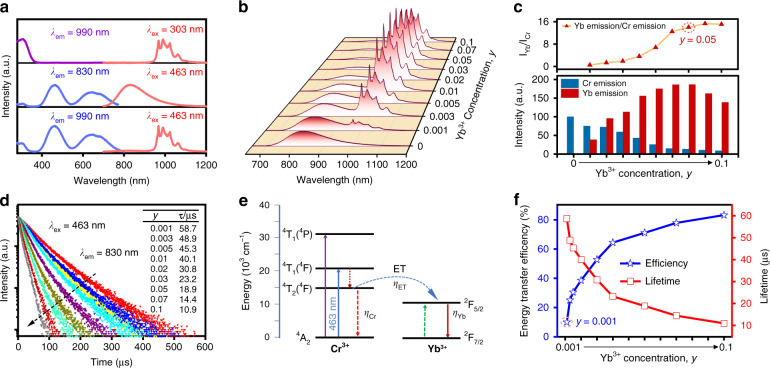
SWIR luminescence properties of Lu_0.2_Sc_0.8_BO_3_:Cr^3+^,Yb^3+^ phosphors. **a** Photoluminescence emission and excitation spectra of Lu_0.2_Sc_0.8_BO_3_:5%Yb^3+^, Lu_0.2_Sc_0.8_BO_3_:2%Cr^3+^, and Lu_0.2_Sc_0.8_BO_3_:2%Cr^3+^,5%Yb^3+^ phosphors. **b** Photoluminescence emission spectra of Lu_0.2_Sc_0.8_BO_3_:2%Cr^3+^,*y*Yb^3+^ (*y* = 0–0.1) upon 463-nm-light excitation. **c** The ratio of Yb^3+^ (940–1200 nm) SWIR emission to Cr^3+^ NIR emission (650–940 nm), and Cr^3+^ NIR emission intensity and Yb^3+^ SWIR emission intensity in Lu_0.2_Sc_0.8_BO_3_:2%Cr^3+^,*y*Yb^3+^ phosphors with varying Yb^3+^ doping concentration. **d** Luminescence decay curves of Cr^3+^ in Lu_0.2_Sc_0.8_BO_3_:2%Cr^3+^,*y*Yb^3+^ phosphors monitored at 830 nm with different Yb^3+^ concentration. **e** Schematic representation of Cr^3+^→Yb^3+^ energy transfer. **f** The measured Cr^3+^ lifetime and Cr^3+^→Yb^3+^ energy transfer efficiency at different Yb^3+^ concentration

The photoluminescence emission and excitation spectra of Lu_0.2_Sc_0.8_BO_3_:5%Yb^3+^, Lu_0.2_Sc_0.8_BO_3_:2%Cr^3+^ and Lu_0.2_Sc_0.8_BO_3_:2%Cr^3+^,5%Yb^3+^ phosphors are shown in Fig. 3a. For Yb^3+^ single-doped phosphor, the excitation spectrum when monitored at 990 nm emission contains one excitation band centered at 303 nm, originating from the absorption of Yb^3+^-O^2−^ charge transfer state^58,59^. When Yb^3+^ is introduced into Lu_0.2_Sc_0.8_BO_3_:2%Cr^3+^ phosphors in an appropriate concentration, strong SWIR emission in a wavelength range over 940–1200 nm that is associated with the characteristic ^2^F_5/2_ → ^2^F_7/2_ transition of Yb^3+^ is observed upon 463-nm-light excitation. The excitation spectrum of Lu_0.2_Sc_0.8_BO_3_:2%Cr^3+^,5%Yb^3+^ phosphor monitored at 990 nm is in accordance with that of Lu_0.2_Sc_0.8_BO_3_:2%Cr^3+^, suggesting the emergence of efficient Cr^3+^→Yb^3+^ energy transfer. The strong absorption in the blue region for Lu_0.2_Sc_0.8_BO_3_:Cr^3+^,Yb^3+^ phosphor (Fig. S8) allows high-efficiency blue-to-SWIR conversion to be realized when combined with commercial high-power blue LEDs.

The emission spectra of Lu_0.2_Sc_0.8_BO_3_:2%Cr^3+^,*y*Yb^3+^ with varying Yb^3+^ concentration when excited at 463 nm are shown in Fig. 3b. Note that the variation of Yb^3+^ content induces a noticeable change in the emission spectra, that is, SWIR emission band over 940–1200 nm peaking at around 1000 nm begins to appear and finally dominates the emission spectra with the increase of Yb^3+^ concentration. As presented in Fig. 3c, significant enhancement of SWIR luminescence and overall emission are detected with increasing Yb^3+^ concentration over 0–0.1. The optimal doping level of Yb^3+^ is determined to be 0.05, at which the overall integrated intensity increases by two times in comparison with that of Lu_0.2_Sc_0.8_BO_3_:2%Cr^3+^ phosphor and the Cr^3+^ NIR emission accounts for only around 6.7% of the total emission. Even at *y* = 0.1, the total integrated emission intensity is still significantly higher than Lu_0.2_Sc_0.8_BO_3_:Cr^3+^ phosphor, and the proportion of Cr^3+^ emission further decreases. More importantly, the absolute quantum yield of Lu_0.2_Sc_0.8_BO_3_:2%Cr^3+^,5%Yb^3+^ phosphor has been improved to 73.6% because of the intense SWIR emission of Yb^3+^ (Fig. S7b). Note that Yb^3+^ infrared emission based on the Cr^3+^→Yb^3+^ energy transfer has also been observed in other material systems^40–42^. For example, by co-doping Yb^3+^ into Ca_2_LuZr_2_Al_3_O_12_:Cr^3+^ phosphor and adjusting the Yb^3+^ concentration, He et al. achieved super broadband NIR luminescence with a large FWMH of 320 nm and a high absolute quantum yield of 77.2%^40^. Yao et al. demonstrated that the NIR photoluminescence performance of LiScP_2_O_7_:Cr^3+^ phosphor can be greatly improved after co-doping Yb^3+,^^41^. However, the ratio of Yb^3+^ emission to Cr^3+^ emission in LiScP_2_O_7_:6%Cr^3+^,3%Yb^3+^ is only about 7.9, while the ratio is determined to be ~14 in Lu_0.2_Sc_0.8_BO_3_:2%Cr^3+^,5%Yb^3+^, indicating that the Cr^3+^ emission is greatly reduced in the composition of total emission and more pure SWIR emission is obtained in this phosphor. Additionally, the LiIn_2_SbO_6_:Cr^3+^,Yb^3+^ phosphor reported by Liu et al.^42^ exhibits very low internal quantum efficiency (10%) and poor thermal stability (31%@95 °C) upon 492 nm excitation.

The luminescence decay curves of Lu_0.2_Sc_0.8_BO_3_:2%Cr^3+^,*y*Yb^3+^ with different Yb^3+^ concentrations by monitoring 830 nm emission when excited by blue light at 463 nm are depicted in Fig. 3d, the lifetimes of Lu_0.2_Sc_0.8_BO_3_:2%Cr^3+^,*y*Yb^3+^ phosphors show a significant declining trend from 58.7 to 10.9 μs as the content of Yb^3+^ increases, further verifying the appearance of efficient Cr^3+^→Yb^3+^ energy transfer, as depicted in Fig. 3e. Considering that the energy mismatch between Cr^3+^ donor and Yb^3+^ acceptor can be compensated by the emission of phonons, phonon-assisted Cr^3+^→Yb^3+^ energy transfer will be responsible for the SWIR emission under the excitation of blue light^60^. The efficiency of energy transfer (*η*_*ET*_) is determined by the following equation^61,62^:

5 $$\eta _{ET} = 1 - \left( {\tau /\tau _0} \right)$$

where *τ* and τ_0_ are the Cr^3+^ lifetimes with and without Yb^3+^, respectively. The luminescence lifetime and the calculated efficiency of energy transfer at varied concentration of Yb^3+^ are presented in Fig. 3f. The maximum energy transfer efficiency can reach up to 84.2% for Lu_0.2_Sc_0. 8_BO_3_:2%Cr^3+^,10%Yb^3+^ phosphor.

**Fig. 4:**
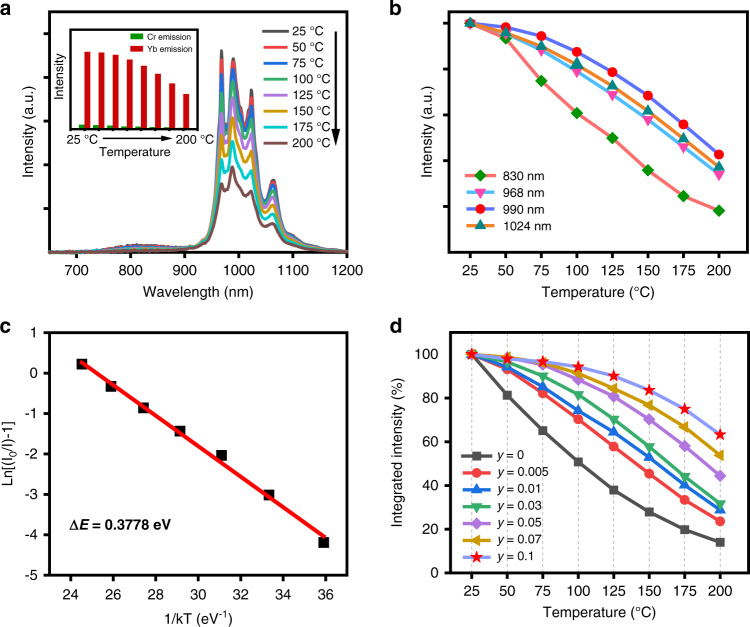
Temperature-dependent photoluminescence properties of Lu_0.2_Sc_0.8_BO_3_:Cr^3+^,Yb^3+^ phosphors. **a** Emission spectra of Lu_0.2_Sc_0.8_BO_3_:2%Cr^3+^,5%Yb^3+^ at different temperature. Inset: variation of the Cr^3+^ emission intensity and Yb^3+^ emission intensity. **b** The peak wavelength intensities (830, 968, 990, and 1024 nm) of Lu_0.2_Sc_0.8_BO_3_:2%Cr^3+^,5%Yb^3+^ at varied temperature. **c** The fitting drawn in Ln(*I*/*I*_0_-1) *versus* 1/*kT* coordinates. **d** Dependence of integrated luminescence intensity on the test temperature with varying Yb^3+^-doping concentration

Environment temperature has a great effect on the photoluminescence performance of luminescent materials, thereby further affecting the luminescence properties of the fabricated photoelectric devices. As a result, the luminescence thermal stability of the yielded SWIR emissive material is also investigated in detail. The emission spectra of Lu_0.2_Sc_0.8_BO_3_:2%Cr^3+^,5%Yb^3+^ phosphor at different temperature over 25–200 °C are shown in Fig. 4a. Along with increasing the test temperature, the drop in overall luminescence intensity is detected due to the increase of nonradiative transition probability. The NIR luminescence of Cr^3+^ almost disappears at elevated temperature, while Yb^3+^ luminescence intensity remains at a relatively high level (inset in Fig. 4a). Meanwhile, the variation trend of different peak emission intensities (830, 968, 990, and 1024 nm) of Lu_0.2_Sc_0.8_BO_3_:2%Cr^3+^,5%Yb^3+^ phosphor is different (Fig. 4b). The 990 nm emission peak of Yb^3+^ shows the weakest thermal quenching behavior, which results in the redshift of the Yb^3+^ SWIR emission peak from 968 nm to 990 nm as the temperature rises. When the temperature is increased to 100 °C, 88.4% of the initial luminescence intensity at 25 °C can be retained for Lu_0.2_Sc_0.8_BO_3_:2%Cr^3+^,5%Yb^3+^. The Arrhenius equation can be used to calculate the activation energy (∆*E*), as shown below^63,64^:

6 $$I_T = \frac{{I_0}}{{1 + A\exp \left( {\frac{{ - \Delta E}}{{kT}}} \right)}}$$

where *I*_*T*_ is the intensity of emission at a given temperature *T*, *I*_0_, *k,* and *A* are constants, Δ*E* refers to the activation energy, which corresponds to the energy gap from the lowest position of the excited level to the cross point of the excitation level and the ground state level. The fitting is plotted as the red curve in Fig. 4c. Using *I*_*0*_ = *I*(25 °C), the Δ*E* value is determined to be 0.38 eV, which is consistent with the superior luminescence thermal stability of Lu_0.2_Sc_0.8_BO_3_:2%Cr^3+^,5%Yb^3+^ phosphor. In addition, emission spectra of Lu_0.2_Sc_0.8_BO_3_:2%Cr^3+^,*y*Yb^3+^ phosphors at different temperatures with varying Yb^3+^ concentrations are also provided in Fig. S9. Figure 4d depicts the integrated luminescence intensity of Lu_0.2_Sc_0.8_BO_3_:2%Cr^3+^,*y*Yb^3+^ phosphors at varied temperatures. Compared with Cr^3+^ single-doped phosphor (50%@100 ºC), the luminescence thermal stability shows an obvious increase as Yb^3+^ concentration increases, suggesting that Yb^3+^ emission is more thermally stable than Cr^3+^ emission. It should also be noted that the thermal quenching of Cr^3+^ and Cr^3+^→Yb^3+^ energy transfer are competitive in Cr^3+^-Yb^3+^-co-doped material system. The excitation energy will be transferred to thermally stable Yb^3+^ emitters rapidly to suppress luminescence thermal quenching of the excited Cr^3+^ ions. With the increase of Yb^3+^ content, SWIR luminescence of Yb^3+^ through energy transfer from Cr^3+^ dominates the total emission.

### Luminescence properties of phosphor-converted SWIR emitters

**Fig. 5:**
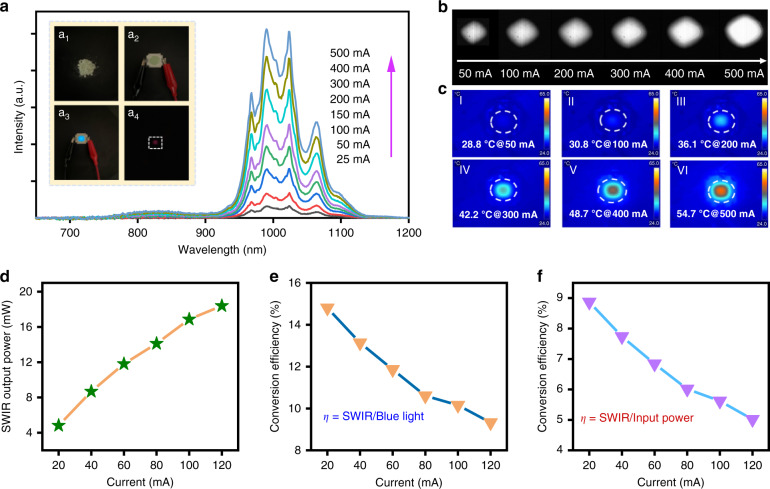
Luminescence properties of phosphor-converted SWIR light sources. **a** Emission spectra of the SWIR LED through a combination of Lu_0.2_Sc_0.8_BO_3_:2%Cr^3+^,5%Yb^3+^ with blue LED at varied current over 25–500 mA. The upper insets show the as-synthesized phosphor powders (a_1_), the constructed high-power SWIR LED prototype device (a_2_), working SWIR LED taken by a standard digital camera in the absence (a_3_) and presence (a_4_) of a long-pass filter at 650 nm. **b** SWIR images of the prototype device at varied driving currents. **c** Thermal images and actual working temperatures of SWIR LED device at different input currents. **d** SWIR output power of the fabricated LED with varying the input current. **e**, **f** Blue-to-SWIR power conversion efficiency (the ratio of SWIR radiant power to the output power of blue LED) and electricity-to-SWIR light energy conversion efficiency (the ratio of SWIR radiant power to the input electrical power) of the SWIR LED as a function of the driving current

Inspired by the appealing ability of Lu_0.2_Sc_0.8_BO_3_:Cr^3+^,Yb^3+^ phosphor to efficiently convert blue photons into SWIR luminescence, we successfully developed SWIR LED prototype devices through a combination of blue InGaN chip and our SWIR-emitting phosphor. The as-synthesized phosphor powders (a_1_), the fabricated high-power SWIR LED prototype device (a_2_), a working SWIR LED taken by a standard digital camera in the absence (a_3_), and presence (a_4_) of a long-pass filter at 650 nm are shown in Fig. 5a (insets). Blue light emission from the internal InGaN LED can be noticed when the LED is lit, which can be fully blocked by the long-pass filter. The emission spectra of SWIR LED prototype device under different input currents are displayed in Fig. 5a. The constructed LED device exhibit a dominant SWIR emission band over 940–1200 nm, which evidences the conversion efficiency from blue to SWIR by the phosphor layer is very efficient. With a rise of the injection current over 25–500 mA, the SWIR LED shows an increasing trend in emission intensity (Fig. 5a, b). Meanwhile, a thermal imager is employed to record the temperature changes of the SWIR LED when it is in operation, as shown in Fig. 5c. The actual operating temperature increases very slowly from 28.8 to 54.7 °C when the driving current increases over 50–500 mA. The small temperature variation of the prototype device during operation indicates that less input electric energy is being converted to heat, suggesting that the fabricated phosphor-converted prototype device is a prospective high-power SWIR light source for numerous scientific and technological applications. Besides, the fabricated SWIR LED has a power output capacity of 18.4 mW at 120 mA (~3.1 V), as presented in Fig. 5d. As the input current increases in the 20–120 mA, the blue-to-SWIR conversion efficiency decreases from 14.8% to 9.3% and the electricity-to-SWIR conversion efficiency drops from 8.9% to 5.0% (Fig. 5e, f), which can be attributed to the efficiency drop of the inside blue LED (Fig. S10). Overall, the resulting prototype device exhibits exceptional output power and efficiency for phosphor-converted SWIR LED with a peak maximum at around 1000 nm, and its luminescence performance will be improved further by the optimization of the LED fabrication.

### Applications of the fabricated SWIR LEDs

**Fig. 6:**
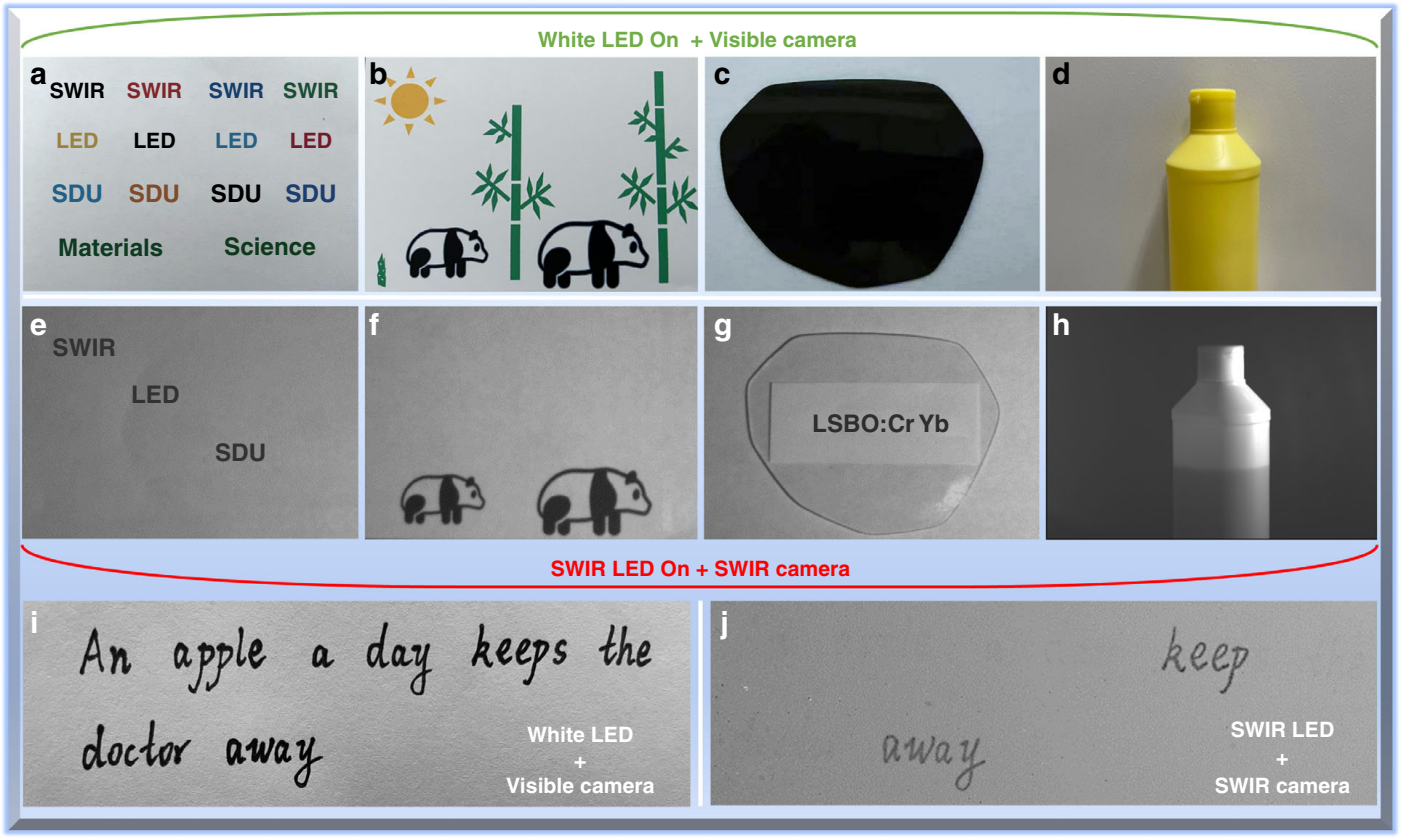
Applications based on the SWIR LED prototype device. **a**–**d** Visible images taken with a standard digital camera when illuminated with white LED. **e**–**h** SWIR images taken with an InGaAs SWIR camera when the SWIR LED is turned on. **i**, **j** Photographs of a short message written by black carbon pen and black watercolor pen when illuminated with white LED and SWIR LED, respectively

Given the favorable characteristics of SWIR light, a series of new and exciting applications have been designed and demonstrated, as shown in Fig. 6. The colorful English words and stick figure of pandas can be detected clearly by a standard digital camera under white LED illumination (Fig. 6a, b), while nothing can be observed with the aid of the SWIR camera (Fig. S11) because it is not sensitive to the indoor visible light (spectral response range: 900–1700 nm). In contrast, by applying the fabricated SWIR LED to replace the white LED as a lighting source, only the black text and two black pandas can be observed with the help of the SWIR camera, while the colored words, the green bamboo, and the yellow sun disappear completely on the pictures (Fig. 6e, f). It is known that the black texts and graphics contain large amounts of black carbon, which has strong absorption of SWIR light, while other colored texts and graphics without carbon weakly absorb SWIR light. Therefore, the colored paints can be penetrated by the SWIR light, enabling them to show the same grayscale color with the background, while their counterparts containing black carbon strongly absorb SWIR light and do not create reflections, resulting in very low grayscale value. Moreover, much like X-rays, SWIR light has the potential to provide imaging through different materials by virtue of its exceptional penetration ability with respect to light in the UV-to-visible wavelength range. As shown in Fig. 6g, the English message “LSBO: Cr Yb” covered by dark sunglasses can be easily and clearly observed under the illumination of SWIR LED when used in conjunction with an InGaAs camera, while this message is invisible when illuminated with white LED light because of the protection of dark sunglasses (Fig. 6c). The SWIR light from the prototype device is also used to determine the fill level of an opaque plastic detergent bottle, which is able to produce high contrast images for precise non-constructive monitoring, as shown in Fig. 6d, h. Next, we took a step further to test the fabricated SWIR device to accurately and quickly distinguish key information when used together with a SWIR camera. As shown in Fig. 6i, a short message was written on the white paper, in which the real information was written in carbon pen and the interference message was written in black watercolor pen. They are exactly the same to the naked eye when illuminated with white LED. However, under the illumination of SWIR LED, the right information that is carbon-rich can be read clearly by using the SWIR camera, as shown in Fig. 6j. Other typical applications for night vision technology and non-destructive detection are also presented (Fig. S12). Furthermore, NIR LED based on Lu_0.2_Sc_0.8_BO_3_:Cr^3+^ can not realize the same applications due to the lack of adequate SWIR light in its emission spectrum (Fig. S13). The outcomes of this study strongly demonstrate the great potential of the synthesized Lu_0.2_Sc_0.8_BO_3_:Cr^3+^,Yb^3+^ SWIR-emitting phosphor as an excellent luminescence converter for high-performance SWIR light source.

## Discussion

In conclusion, highly efficient and thermally stable SWIR-emitting Lu_0.2_Sc_0.8_BO_3_:Cr^3+^,Yb^3+^ phosphors suitable for the excitation of high-power blue LED have been designed and developed. Not only can these phosphors accommodate high content of Yb^3+^, resulting in dominant SWIR photoluminescence, but they also are comparatively easy to make and air-stable once formed. Upon excitation with 463 nm blue light, the as-synthesized Lu_0.2_Sc_0.8_BO_3_:Cr^3+^,Yb^3+^ phosphor yields intense SWIR emission peaking at around 1000 nm with an absolute photoluminescence quantum yield of ~73.6%. Moreover, its emission intensity at 100 °C can maintain 88.4% of the initial value at 25 °C. On this basis, a high-performance SWIR LED prototype device emitting at ~1000 nm is successfully constructed through a combination of the optimized Lu_0.2_Sc_0.8_BO_3_:2%Cr^3+^,5%Yb^3+^ phosphor and blue LED chip, delivering an optical power of 18.4 mW with 9.3% of blue-to-SWIR power conversion efficiency and 5.0% of electricity-to-SWIR light energy conversion efficiency at 120 mA. Compared to conventional phosphor-converted white LEDs, whose application is constrained to general illumination, phosphor-converted SWIR illuminators here can give access to numerous striking applications when used in conjunction with a commercial SWIR camera, including non-destructive inspection, chemical analysis, anti-counterfeiting and night vision lighting. Overall, this study will lay the groundwork to develop new categories of high-efficient phosphor-converted SWIR LEDs and help bring more awareness to the benefits and solutions of SWIR technology.

## Materials and methods

### Synthesis

Lu_1−x_Sc_x_BO_3_:Cr^3+^ and Lu_0.2_Sc_0.8_BO_3_:Cr^3+^,Yb^3+^ phosphors were prepared via a conventional solid-state reaction method. Lu_2_O_3_ (Aladdin, 99.99%), Sc_2_O_3_ (Aladdin, 99.99%), H_3_BO_3_ (Aladdin, 99.99%), Cr_2_O_3_ (Aladdin, 99.99%), and Yb_2_O_3_ (Aladdin, 99.99%) were used as the starting precursors and ground adequately, except that 25% excess of H_3_BO_3_ was added to compensate for the evaporation loss of boron element. After that, the obtained mixture was transferred into a muffle furnace and sintered at different temperatures over 1150–1300 °C, and kept for 5 h in the air to form the final products.

### Fabrication of SWIR LED prototype device

The optimized Lu_0.2_Sc_0.8_BO_3_:2%Cr^3+^,5%Yb^3+^ phosphor and blue SMD 2835 LED (452.5−455 nm) were integrated to construct SWIR LED prototype. The phosphor powder and high refractive index encapsulation silicone glue (BQ-8229A/B, Betterly New Materials Co.,Ltd) were thoroughly mixed in a mass ratio of 1:1 and then deposited on the blue LED chips. The phosphor coating thickness is about 0.3 mm. High-power SWIR LED prototype was also constructed through a combination of Lu_0.2_Sc_0.8_BO_3_:2%Cr^3+^,5%Yb^3+^ phosphor with high-power blue LED (LEDGUHON, 460–465 nm).

### Characterization

XRD was collected on a DMAX-2500PC X-ray diffractometer. The microstructure and elemental composition of the as-synthesized phosphor were measured on a scanning electron microscope (SEM, JSM-7800F). The phosphor powder was deposited on an aluminum foil substrate for measurement. The element composition and distribution were further identified by inductively coupled plasma mass spectrometry (ICP-MS). UH4150 spectrophotometer was used to measure the diffuse reflection spectra. The photoluminescence measurements of the yielded phosphors and the luminescence properties of the fabricated SWIR LEDs were measured using similar test equipment to that described in our previous work^32^. Utmost care was taken to assure the reproducibility of the spectral measurements. A thermal imaging camera (FLIR A300) was used to record the operating temperature of the SWIR LEDs. A Canon EOS 800D digital camera and a commercial InGaAs SWIR camera (Raptor Owl 640 S) were used to capture visible and SWIR images, respectively.

## Supplementary information

Supplemental material
