# Supplementary material for: Blue LED-pumped intense short-wave infrared luminescence based on Cr3+-Yb3+-co-doped phosphors
Source: Light Sci Appl. 2022 May 13;11:136. doi: 10.1038/s41377-022-00816-6 (PMC9106724; doi:10.1038/s41377-022-00816-6)
Supplement: Supplementary file 1 — Supplemental material [file 41377_2022_816_MOESM1_ESM.pdf]

Supporting Information

Blue LED-pumped intense short-wave infrared luminescence based on Cr^{3+}-Yb^{3+}-codoped phosphors

Yan Zhang[1†], Shihai Miao[1†], Yanjie Liang[1]*, Chao Liang[2], Dongxun Chen[1], Xihui Shan[1], Kangning Sun[1], Xiao-Jun Wang[3]*

[1]Key Laboratory for Liquid-Solid Structure Evolution and Processing of Materials, Ministry of Education, Shandong University, Jinan 250061, China

[2]Jiangsu Bree Optronics Co., Ltd., Nanjing 211103, China

[3]Department of Physics, Georgia Southern University, Statesboro, GA 30460, USA

[†]These authors contributed equally to this work.

Correspondence: Yanjie Liang (yanjie.liang@sdu.edu.cn)

Xiao-Jun Wang (xwang@georgiasouthern.edu)

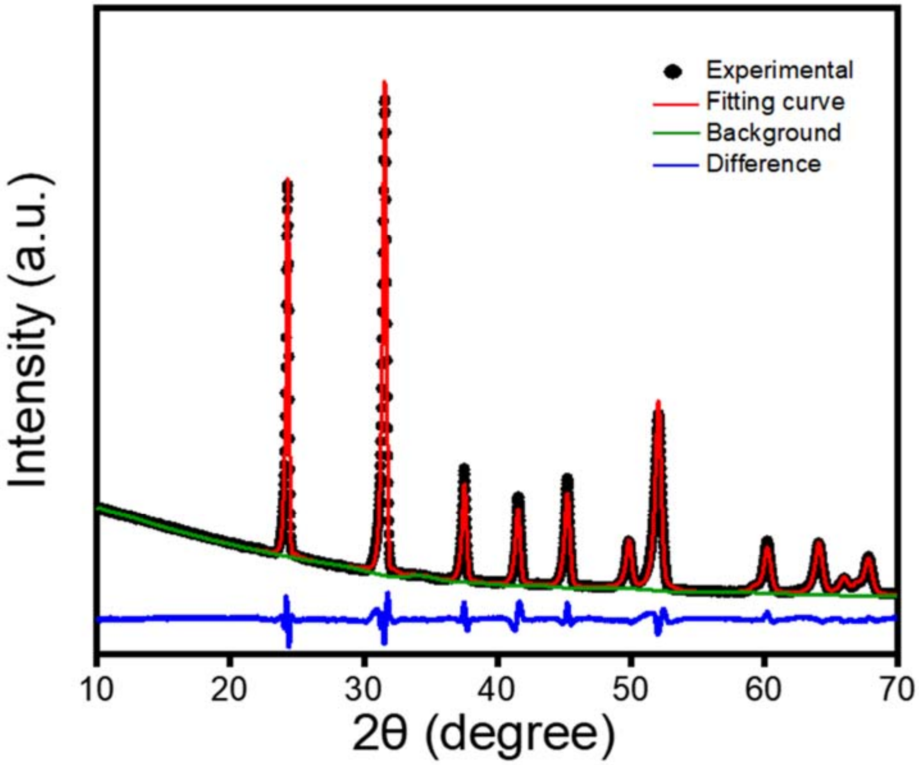

Fig. S1 Rietveld refinement of $Lu_{0.2}Sc_{0.8}BO_3$:2%Cr^{3+},5%Yb^{3+} phosphor.

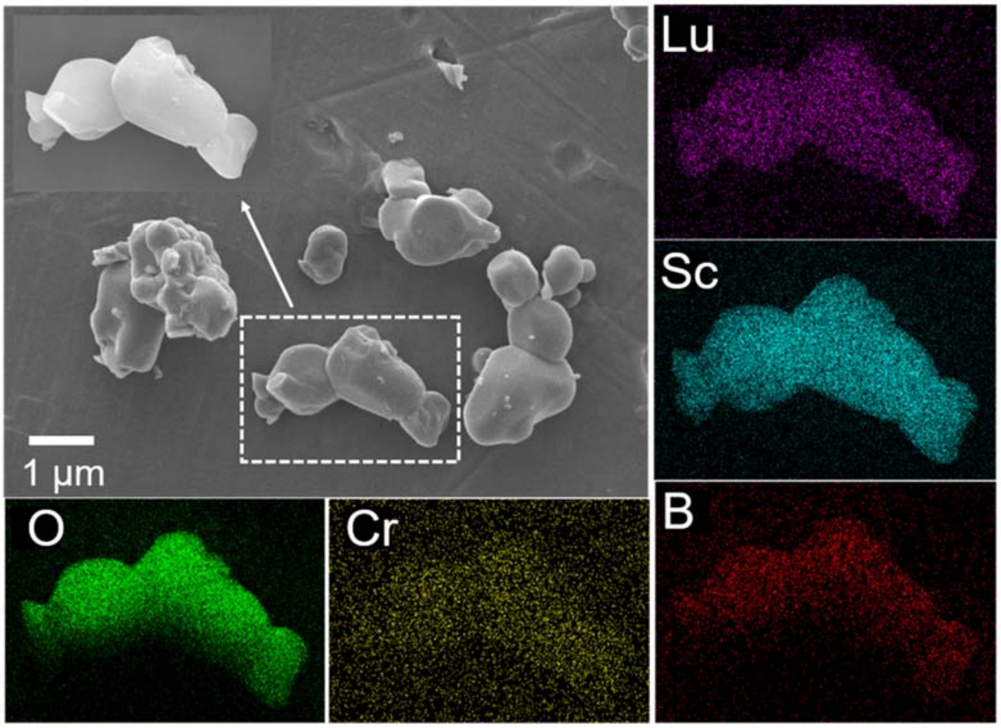

Fig. S2 SEM and EDS elemental mapping images of the $Lu_{0.2}Sc_{0.8}BO_3$:Cr^{3+} phosphor.

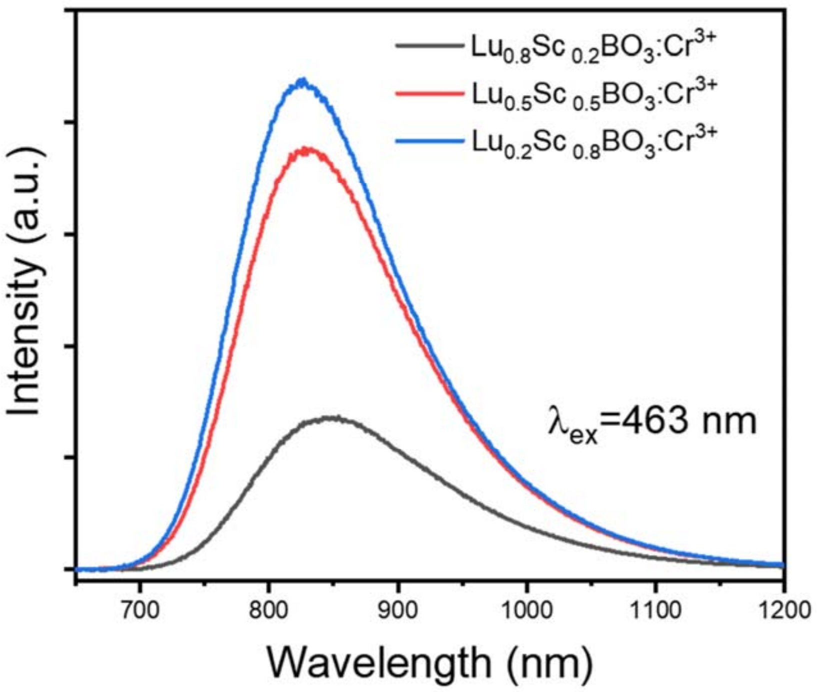

Fig. S3 Photoluminescence emission spectra of $Lu_{1-z}Sc_zBO_3:Cr^{3+}$ phosphors (z = 0.2–0.8) at room temperature.

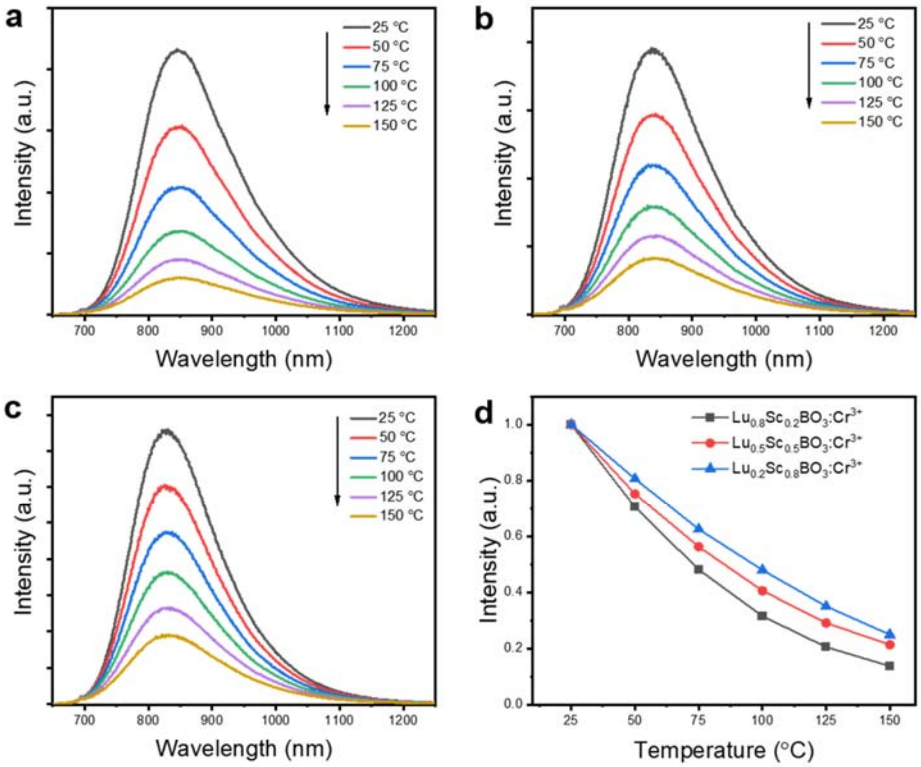

Fig. S4 (a-c) Temperature-dependent emission spectra of $Lu_{1-z}Sc_zBO_3:Cr^{3+}$ phosphors (z = 0.2–0.8). (d) Dependence of the emission intensity on the temperature of the above three samples.

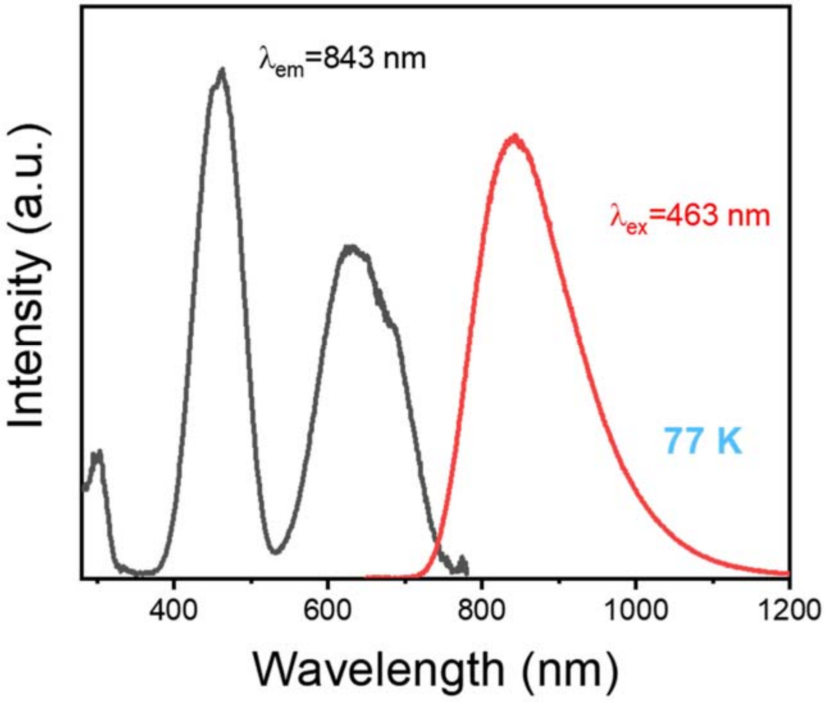

Fig. S5 Photoluminescence emission and excitation spectra of $Lu_{0.2}Sc_{0.8}BO_3:Cr^{3+}$ phosphor at 77 K.

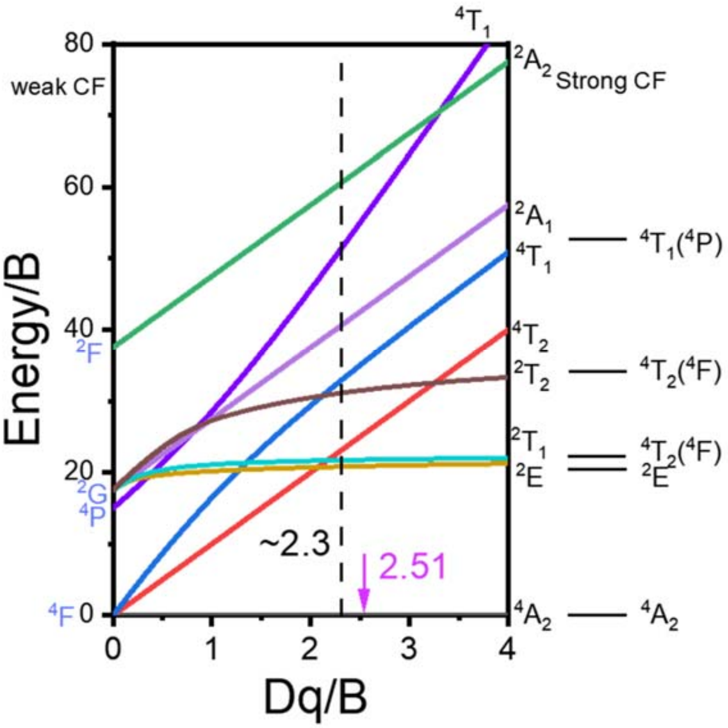

Fig. S6 Tanabe–Sugano energy level diagram for Cr^{3+} ($3d^3$) ions in the octahedral crystal field of $Lu_{0.2}Sc_{0.8}BO_3:Cr^{3+}$ compounds.

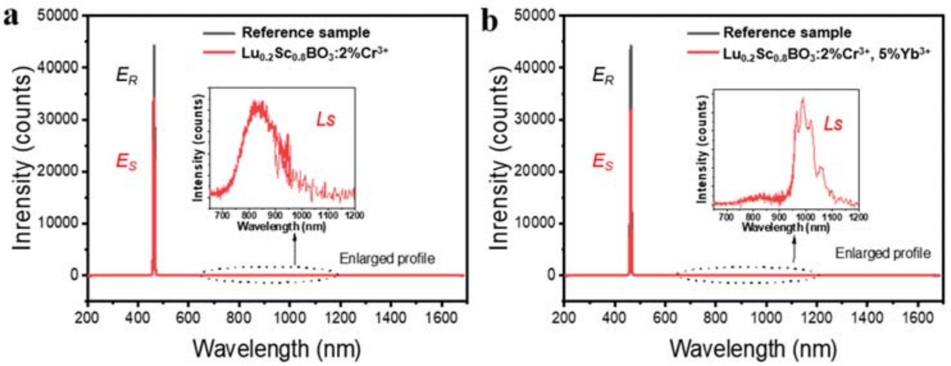

Fig. S7 (a, b) Excitation line of $BaSO_4$ and emission spectrum of the $Lu_{0.2}Sc_{0.8}BO_3$:2%Cr^{3+} and $Lu_{0.2}Sc_{0.8}BO_3$:2%Cr^{3+},5%Yb^{3+} phosphors collected using an integrating sphere. The inset shows a magnification of the emission spectrum.

As shown in Fig. S7, the internal QE value can be calculated by the following equation:

$$\eta_{QE} = \frac{\int L_S}{\int E_R - \int E_S} \qquad (1)$$

where E_R is the spectrum of the excitation light without the sample in the sphere, E_S is the spectrum of the light used for exciting the sample, and L_S is the emission spectrum of the studied sample. The measured internal quantum efficiencies of $Lu_{0.2}Sc_{0.8}BO_3$:2%Cr^{3+} and $Lu_{0.2}Sc_{0.8}BO_3$:2%Cr^{3+},5%Yb^{3+} phosphors are determined to be 26.1% and 73.6%, respectively.

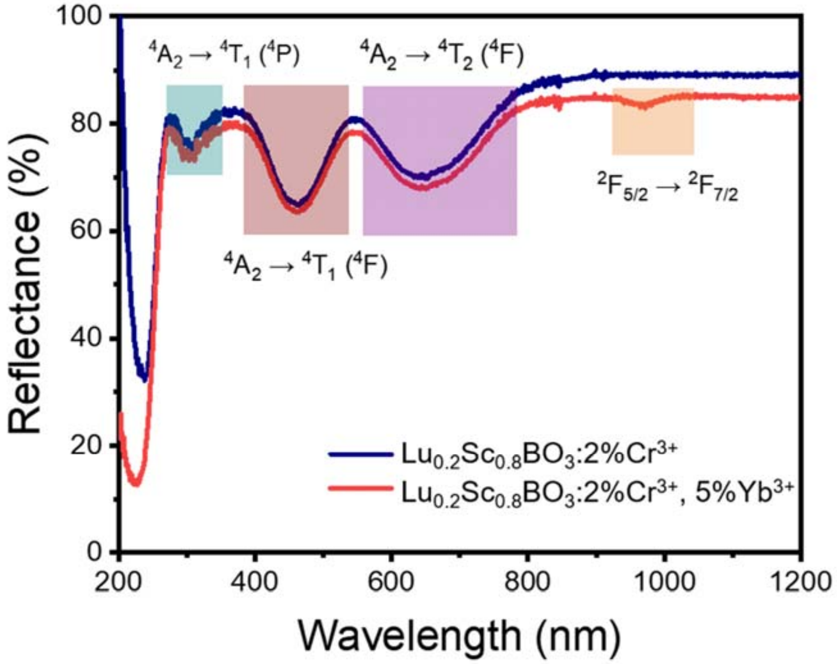

Fig. S8 Diffuse reflectance spectra of $Lu_{0.2}Sc_{0.8}BO_3$:2%Cr^{3+} and $Lu_{0.2}Sc_{0.8}BO_3$:2%Cr^{3+},5%Yb^{3+} phosphors.

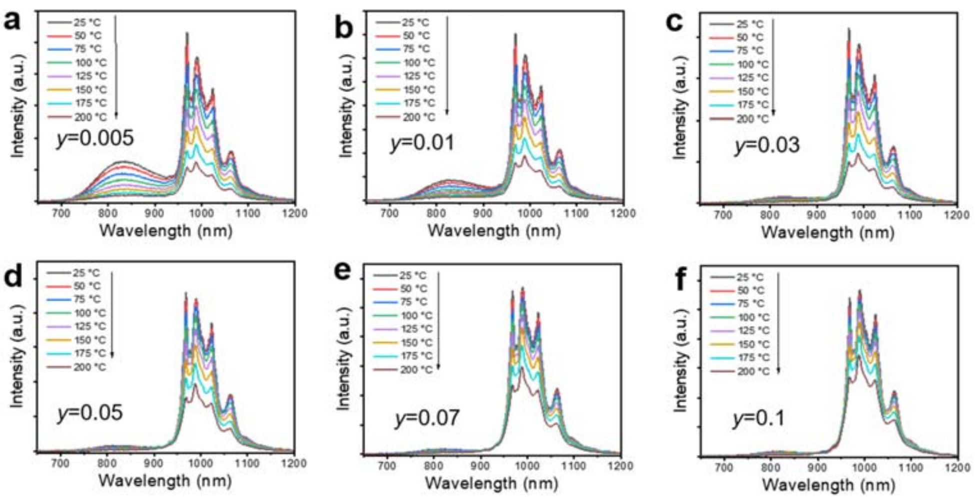

Fig. S9 Temperature-dependent emission spectra of $Lu_{0.2}Sc_{0.8}BO_3$:2%Cr^{3+},$y$$Yb^{3+}$ phosphors (y = 0.005–0.1).

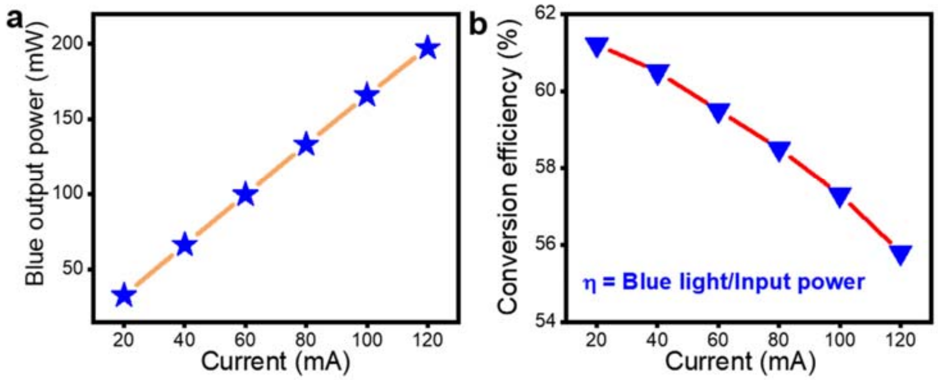

Fig. S10 (a) Output power of blue LED chip under various drive currents. (b) The power conversion efficiency of blue LED chip as a function of drive currents.

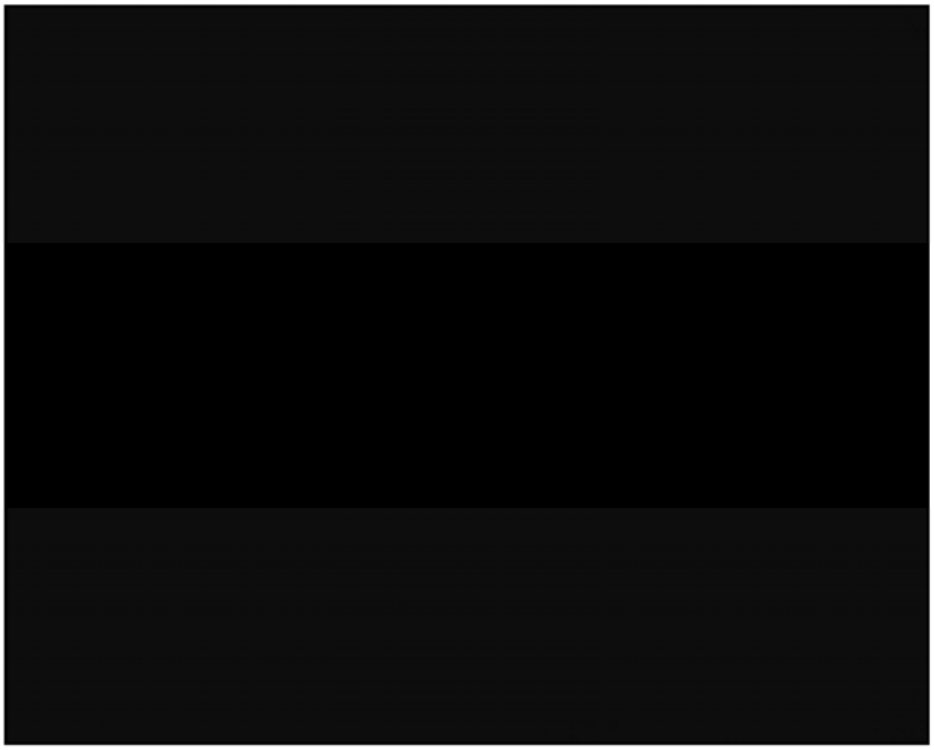

Fig. S11 Colorful English words and stick figure of pandas detected by the SWIR camera under the illumination of white LED.

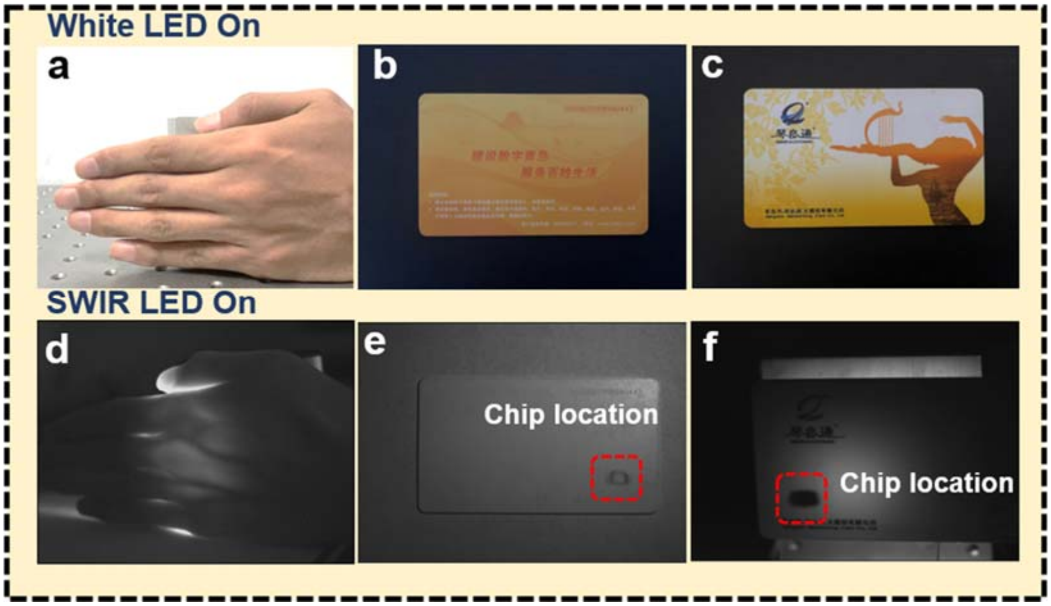

Fig. S12 (a-c) Visible images taken with a standard camera in indoor lighting (white LED) environment. (d-f) SWIR images taken with a SWIR camera when the SWIR LED is turned on.

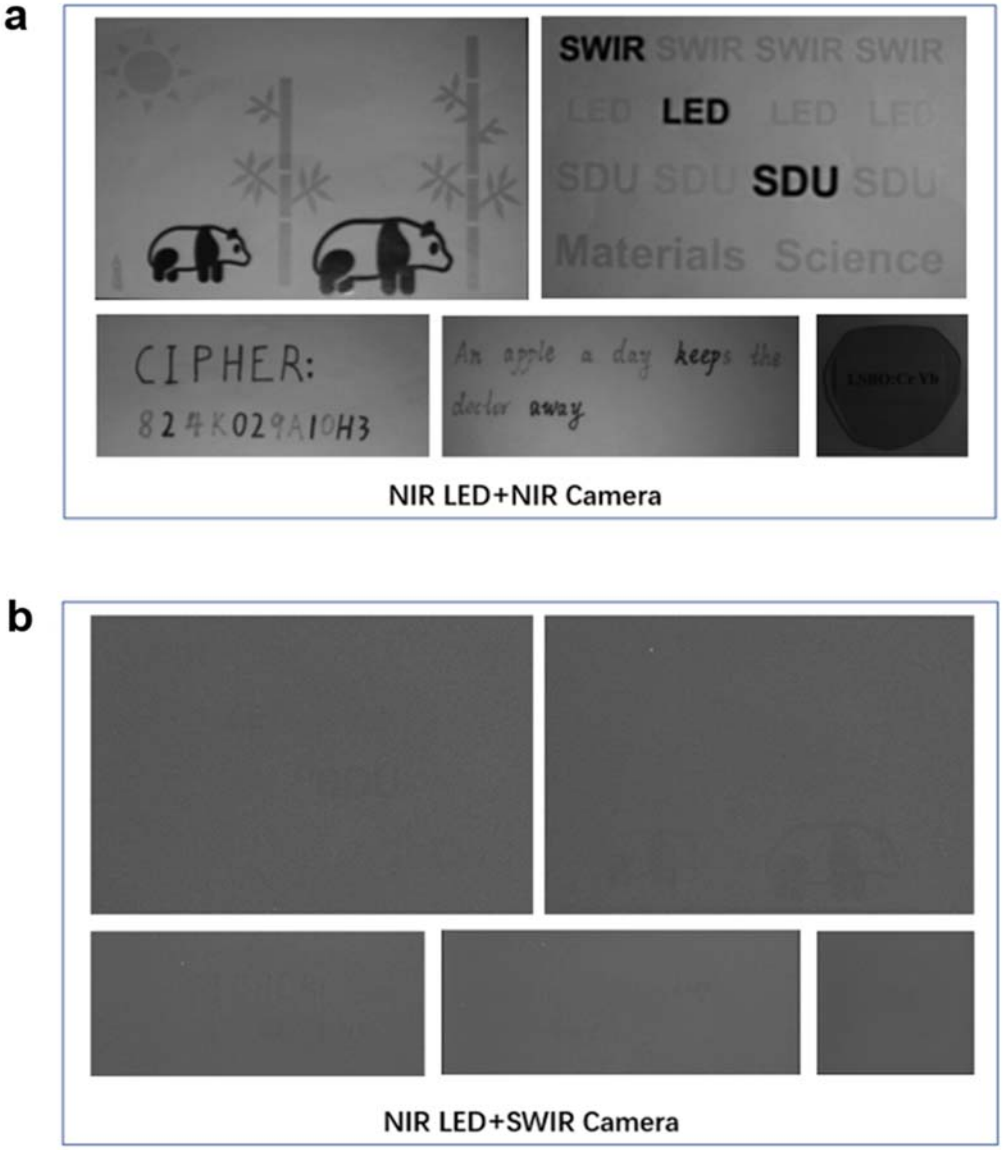

Fig. S13 Night vision lighting and covert information identification applications by using phosphor-converted NIR LED. The NIR LED were fabricated by combining blue LED chip with $Lu_{0.2}Sc_{0.8}BO_3$:2%Cr^{3+} phosphor. The Images were taken with an NIR camera (Hikvision MV-CA050-20GN) (a) and an InGaAs SWIR camera (Raptor Owl 640 S) (b), respectively, when the NIR LED is turned on.

Table S1 Refined structural parameters and cell parameter values of $Lu_{0.2}Sc_{0.8}BO_3$:2%Cr^{3+},5%Yb^{3+} from the Rietveld refinement.

Formula	$ScBO_3$		
Crystal system	Rhombohedral		
Space group	$R\bar{3}c$		
Cell parameters	a = 4.7858 Å, b = 4.7858 Å, c = 15.4912 Å		
	Alpha = 90	Beta = 90	Gamma = 120
Cell volume	V =307.2790Å^3		
Z	6		
Reliability factors	R_p = 6.27%, R_{wp} = 8.84%		
Atom	x	y	z
Sc1	0.00000	0.000000	0.000000
B1	0.00000	0.00000	0.25000
O1	0.71622	0.00000	0.25000
Lu1	0.00000	0.00000	0.00000
Cr1	0.00000	0.00000	0.00000
Yb1	0.00000	0.00000	0.00000

Table S2 The ICP analysis of $Lu_{0.2}Sc_{0.8}BO_3$:2%Cr^{3+},5%Yb^{3+} phosphor.

Element	Sample weight/g	Solution volume/ml	Dilution factor	Test Indicating value/mg L^{-1}	Element concentration/ mg kg^{-1}	Element mole fraction/mol%
Lu	0.1475	25	10	6.6229	11225.3169	23.36
Sc	0.1475	25	10	4.914	8328.8134	67.46
Cr	0.1475	25	1	1.2033	203.944	1.43
Yb	0.1475	25	10	2.175	3686.3667	7.76
